# Rare Diseases That Impersonate One Another: X‐Linked Hypophosphatemia and Tumor‐Induced Osteomalacia, a Retrospective Analysis of Discriminating Features

**DOI:** 10.1002/jbm4.10580

**Published:** 2022-01-07

**Authors:** Joseph DeCorte, Ericka Randazzo, Margo Black, Chase Hendrickson, Kathryn Dahir

**Affiliations:** ^1^ Vanderbilt Medical Scientist Training Program Vanderbilt University Medical Center, Vanderbilt University School of Medicine Nashville TN USA; ^2^ Program for Metabolic Bone Disorders at Vanderbilt, Division of Diabetes, Endocrinology, and Metabolism, Department of Medicine Vanderbilt University Medical Center Nashville TN USA; ^3^ Division of Diabetes, Endocrinology, and Metabolism, Department of Medicine Vanderbilt University Medical Center Nashville TN USA

**Keywords:** TUMOR‐INDUCED BONE DISEASE, CANCER, DISORDERS OF CALCIUM/PHOSPHATE METABOLISM, DISEASES AND DISORDERS OF/RELATED TO BONE

## Abstract

Tumor‐induced osteomalacia (TIO) is a rare paraneoplastic disease characterized by frequent fractures, bone pain, muscle weakness, and affected gait. The rarity of TIO and similar presentation to other phosphate‐wasting disorders contribute to a high misdiagnosis rate and long time to correct diagnosis. TIO is curable by tumor resection, so accurate diagnosis has significant impact on patients' emotional and economic burden. Current diagnostics for TIO rely on decades‐old literature with poor phenotypic validation. Here, we identify salient clinical differences between rigorously validated cohorts of patients with TIO (*n* = 9) and X‐linked hypophosphatemia (XLH; *n* = 43), a frequent misdiagnosis for patients with TIO. The TIO cohort had significantly elevated FGF23 (365 versus 95 RU/mL, *p* < 0.001) and alkaline phosphatase (282.8 versus 118.5 IU/L, *p* < 0.01) but significantly reduced phosphorus (1.4 versus 2.2 mg/dL, *p* < 0.05) and 1,25(OH)_2_ D (16.6 versus 59.8 pg/mL, *p* < 0.01). By contrast, total vitamin D was similar between the two groups. Dual‐energy X‐ray absorptiometry (DXA) scans reveal lower *Z*‐scores in the hip (−1.6 versus 0.050, *p* < 0.01) and spine (0.80 versus 2.35, *p* < 0.05). TIO patients were more likely to have prior clinical diagnosis of osteoporosis (67% versus 0%), use assistive devices in daily living (100% versus 14%), and have received a knee arthroplasty (33% versus 7%). TIO patients lost an average of 1.5 cm over their disease course and had sustained an average of 8 fractures each, whereas fractures were rare in XLH. The XLH cohort had higher incidence of osteotomy (19% versus 0%), spinal stenosis (12% versus 0%), secondary dental abnormalities (95% versus 44%, *p* < 0.001), and depression and anxiety (46.5% versus 11%). These results deepen our understanding of the subtle differences present between diseases of phosphate wasting. They suggest several biochemical, clinical, and historical features that effectively distinguish TIO from XLH. © 2022 The Authors. *JBMR Plus* published by Wiley Periodicals LLC on behalf of American Society for Bone and Mineral Research.

## 1 Introduction

Bone metabolism and formation are complex processes mediated by a balance of bone mineralization and bone resorption. A multitude of factors have been identified that can tip this balance to either extreme, resulting in various musculoskeletal deformities and corresponding symptoms. Osteomalacia is the progressive softening of bone due to decreased mineralization of calcium and phosphate at sites of bone remodeling and growth, often accompanied by bone pain and muscle weakness. Of the many hormones involved in bone homeostasis, fibroblast growth factor 23 (FGF23) is a protein secreted from osteocytes mainly in response to serum phosphorus and calcitriol. FGF23 regulates serum phosphorus levels by reducing serum calcitriol, which mediates both renal and intestinal phosphate absorption, and by lowering sodium‐dependent phosphate transport protein 2 (NPT2)‐mediated absorption of phosphate in the kidney. Aberrations in serum FGF23 concentration have been linked to a variety of diseases, such as tumor‐induced osteomalacia (TIO), X‐linked hypophosphatemia (XLH), McCune‐Albright syndrome, and epidermal nevus syndrome.^(^ ^1^, ^2^, ^3^ ^)^

TIO is a rare, paraneoplastic disease characterized by the presence of a phosphaturic mesenchymal tumor (PMT) that secretes excessive amounts of FGF23 into the bloodstream. This leads to osteomalacia via renal phosphate wasting and defective vitamin D metabolism.^(^ ^4^ ^)^ Although TIO is extremely rare in comparison to similar disorders, surgical resection often provides curative treatment, thereby highlighting the necessity of accurate and early diagnosis.^(^ ^5^ ^)^

XLH is a phosphate‐wasting disorder that presents similarly to TIO but is apparently more common. Almost all cases of XLH are caused by mutations in the *PHEX* gene, which expresses the enzyme phosphate‐regulating neutral endopeptidase (PHEX), with a known subset of milder disease caused by mutations in the *PHEX* 3'‐UTR. Previous studies have suggested that the PHEX enzyme may be involved in the regulation of FGF23, a hypothesis that is highlighted by the finding that FGF23 levels are increased or inappropriately normal in XLH patients.^(^ ^6^, ^7^ ^)^

Both XLH and TIO have profound effects on bone health, but the timing of disease onset influences whether bone modeling or remodeling is primarily affected. Because XLH is a genetic disease, the associated mineral deficits often occur throughout the skeletal development of childhood and adolescence, yielding a profound impact on bone modeling. These effects result in the classical picture of rickets, such as bowing deformities and short stature.^(^ ^8^ ^)^ On the other hand, TIO predominantly affects older adults, but cases of TIO in childhood through young adulthood have been reported.^(^ ^9^, ^10^ ^)^ In older adults, epiphyseal growth plates are closed, so that TIO primarily distorts bone remodeling. In children and young adults with TIO, bone modeling is also affected.

As suggested above, although the pathogenic basis of phosphate‐wasting disorders differs widely, this family of disorders often presents similarly in regard to both clinical presentation and radiographic findings. This confounds the diagnosis of phosphate‐wasting disorders based purely on clinical criteria and radiological presentation, leading to frequent initial misdiagnoses. Precise knowledge of the basis of a patient's phosphate‐wasting disorder often allows physicians to tailor management strategies, thus potentially saving several years of hardship for patients. Because of the efficacy of genetic testing, recent emphasis has been placed on using gene panels to accelerate clinicians' diagnosis of phosphate‐wasting disorders.^(^ ^11^, ^12^, ^13^ ^)^

Although genetic paneling has improved diagnostic differentiation, panels are expensive and not possible for all patients. Because FGF23 levels are increased in both XLH and TIO, there exists potential for misdiagnosis of these two disorders.^(^ ^11^ ^)^ Moreover, PMTs are notoriously difficult to identify because of their variability in location and small size, but the potential to cure TIO via their resection emphasizes the need for expedient diagnosis and hence distinguishing features for these two disorders. To date, no study has been performed to identify salient differences between TIO and XLH in adults.^(^ ^4^, ^14^ ^)^

Here, we hypothesize that meaningful variables exist to distinguish adults with TIO and XLH, and we leverage validated databases of adult patients with each disease to retrospectively compare the two.

## 2 Materials and Methods

### 2.1 Patient selection

The study was conducted in accordance with the Declaration of Helsinki and the Good Clinical Practice guidelines developed at the International Conference on Harmonization of Technical Requirements for Registration of Pharmaceuticals for Human Use. We analyzed clinical variables of adult patients with confirmed TIO (*n* = 9) and XLH (*n* = 43). TIO patients were identified in clinic or via the Vanderbilt University Medical Center's identified clinical research database, the Research Derivative. Only patients with an identified PMT and clinical improvement post‐resection were included. XLH patients were identified via the referrals to the Program for Metabolic Bone Disorders at Vanderbilt University Medical Center. All XLH patients in this cohort have a confirmed *PHEX* gene mutation.

### 2.2 The research derivative

Data were taken from the Research Derivative (RD) and Vanderbilt University Medical Center (VUMC). The RD is a database of clinical and related data derived from the VUMC clinical systems and restructured for research. Data are repurposed from VUMC's enterprise data warehouse, which includes data from StarPanel, VPIMS, and ORMIS (Operating Room Management Information System), EPIC, Medipac, and HEO, among others. The medical record number and other person identifiers are preserved within the database. Data types include reimbursement codes, clinical notes and documentation, nursing records, medication data, laboratory data, encounter and visit data, among others. For this project, key study personnel (KSPs) analyzed RD output for patients with TIO and XLH, which includes structured data points, such as ICD 9 or 10 codes and encounter dates; semi‐structured data, such as laboratory tests and results; and unstructured data, such as physician progress reports. The database is maintained by the VUMC Office of Research Informatics under the direction of Paul Harris, PhD.

### 2.3 Data collection and bias limitation

For XLH patients, laboratory data were selected from their first presentation to the clinic before initiation of therapy if possible, but especially before the initiation of XLH‐specific therapies like burosumab, where effects on clinical labs are uncertain. For TIO patients, laboratory data were selected from their first presentation with TIO‐like symptoms (eg, suspicious fracture).

Where available, the following quantitative data were collected: complete blood count (CBC), complete metabolic panel (CMP), lipid panel, thyroid panel, iron panel, urinalysis, urine chemistries, magnesium, phosphate, creatine phosphokinase (CPK), FGF23, vitamin D panel, bone densitometry, and dual‐energy X‐ray absorptiometry DXA scans. Where available, radiology reports for fractures and bone densitometries (eg, reports of *Z*‐scores for DXA scans) were read and included in the results. Where available, the following qualitative data were collected: patient medical history, including past diagnoses of bone‐ and phosphate‐related disorders; patient medication history, with focus on use of calcium, phosphate, and vitamin D therapies; patient surgical history; patient's history of fractures and assistive device use; personal dental history; and family history of relevant bone‐ and phosphate‐related disorders.

Random patients were selected and verified by a second study personnel to ensure validity of data recording.

### 2.4 Missing data

Missing quantitative data were imputed in the context of all other data using missForest, a nonparametric imputation package in R (https://cran.r-project.org/web/packages/missForest/missForest.pdf),^(15^ ^)^ only when >80% of the data were present for each given category. For this study, missForest was used to impute some missing phosphorus, vitamin D/calcitriol, and parathyroid hormone (PTH) measurements (eg, in patients who had a complete metabolic panel but not vitamin D values in their medical records). Insufficient CBC and TmP/GFR data existed for the XLH cohort, so these data were excluded altogether in our analysis. Briefly, missForest trains a random forest algorithm on existing data (continuous and/or categorical), then imputes missing data and calculates imputation error estimates.

### 2.5 Data presentation

Quantitative data are presented in box plots with median and interquartile range (25th and 75th percentiles). All data points are shown unless *n* > 20, in which case individual points (except outliers) are omitted for readability.

### 2.6 Outcomes

Outcomes for each variable examined were defined as significant differences between TIO and XLH patients (see “Statistical analysis”). Relevant non‐outcomes/negative results are also reported for completeness.

### 2.7 Statistical analysis

Baseline continuous variables are presented as medians and interquartile range, as well as maximum and minimum values. Baseline categorical variables are presented as number and percent of population. Baseline characteristics and prevalence will be reported as a whole group. Wilcoxon rank‐sum tests were used to compare continuous outcomes with categorical predictors. Two‐tailed chi‐square tests were used to evaluate categorical outcomes with categorical predictors. Any *p* values <0.05 were considered significant for both these tests and are reported as exact values wherever applicable, except when *p* < 0.001 or *p* > 0.10. Data were analyzed using the R statistical computation software (https://www.r-project.org/).

## 3 Results

### 3.1 Patient characteristics

Nine adult patients with TIO diagnosed via clinical presentation and identification of PMT were included in the present study. The median age of TIO symptom onset was 54 years (range 26 to 67 years, outlier 18 years), and the median time from onset to correct diagnosis was 2 years (range 1 to 8 years, outlier 26 years). PMTs were identified in the brain (*n* = 1), humeral head,^(^ ^1^ ^)^ rib,^(^ ^1^ ^)^ metacarpal,^(^ ^1^ ^)^ hip,^(^ ^2^ ^)^ thigh,^(^ ^1^ ^)^ soleus,^(^ ^1^ ^)^ and tibia.^(^ ^1^ ^)^ TIO was frequently misdiagnosed as osteoporosis (6/9 patients, 67%), XLH (2/9 patients, 22%), and other metabolic bone diseases such as Paget's disease of the bone. As comparators, we identified 43 XLH patients with confirmed PHEX mutations and clinical history consistent with XLH. Accurate description of initial presentation was prohibited by the often lengthy natural history of XLH, but most XLH patients have symptom onset and are diagnosed within the first 2 years of life. A list of demographic information for these patients, as well as a schematic of PMT locations for TIO patients, can be found in Table 1. Beyond the parameters described below, depression and anxiety were more common in XLH than in TIO (46.5% versus 11.1%, *p* = 0.049), potentially linked to ascertainment bias or the longer course of disease in XLH. Interestingly, both TIO and XLH cohorts had elevated median body mass indexes (BMIs) at 30.5 and 30.6 kg/m^2^, respectively (normal range [NR] 18.5 to 24.9 kg/m^2^).

**Table 1:** Summary of the Demographic Information of the Tumor‐Induced Osteomalacia (TIO) and X‐Linked Hypophosphatemia (XLH) Cohorts and Diagram of Tumor Locations in the TIO Cohort

	TIO result (*n* = 9)	XLH result (*n* = 43)
Demographics		
Male/female (*n*)	4:5	12:31
Race and ethnicity	White non‐Hispanic: 7 White Hispanic: 2 Black: 1	White non‐Hispanic: 95% Hispanic: 2.5% Black: 2.5%
Age (years) of presentation,^a^ median (range)	54 (18–67)	37.5 (22, 82)
Years before correct diagnosis, median (range)	2 (1–26)	–
Height (cm), median (range)	167.6 (143.8, 172.7)	154.8 (139.7–175.3)
Decrease from maximum adult height (cm, median + range)	1.5 (0, 6.1)	0
Body mass index (kg/m^2^, median + range)	30.5 (18.0, 48.6)	30.6 (19.5, 47.0)
Common medical and surgical history		
Arthralgia (*n*, %)	5 (56)	16 (37)
Osteoarthritis (*n*, %)	4 (44)	14 (33)
Fractures (*n*)	8 per patient average	5
Pseudofractures (*n*)	0	3 total
Knee arthroplasty (*n*, %)	3 (33)	3 (7)
Hip arthroplasty (*n*, %)	2 (22)	6 (14)
Osteotomy (*n*, %)	0 (0)	8 (19)
Abnormalities in secondary dentition (*n*, %)	4 (44)	33/35 (95)
No. of comorbidities,^b^ median (range)	7 (3–9)	4 (0–12)
Use of assistive devices for daily living (*n*, %)	9 (100)	6 (14)
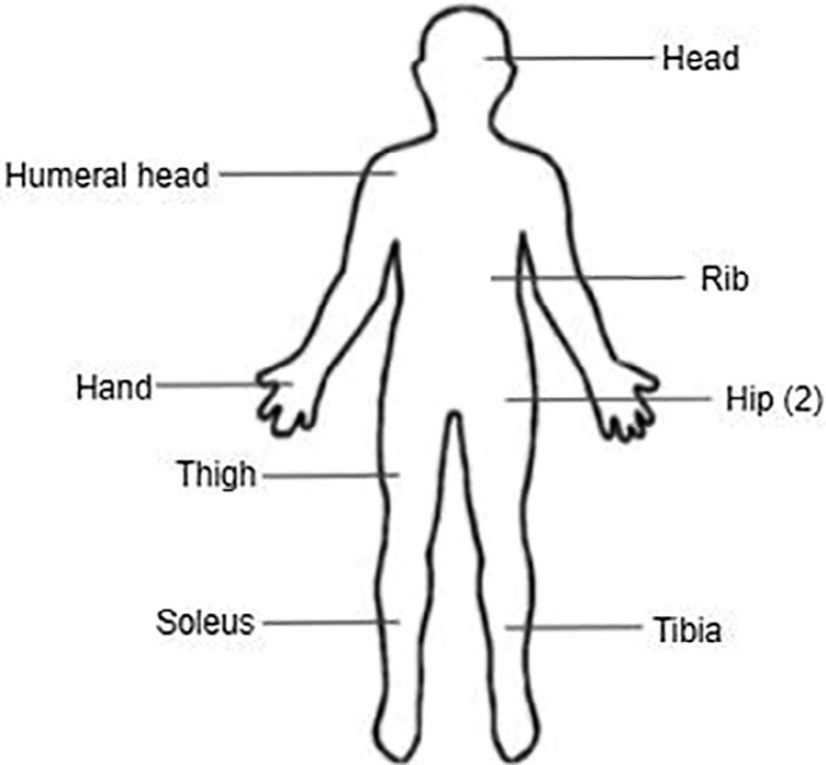		

^a^ Age of presentation indicates when patients presented to our institution for management of symptoms potential/confirmed TIO or XLH, at which time relevant labs and bone scans were performed.

^b^ Comorbidities defined as the number of electronic medical record (EMR)‐linked diagnoses per patient.

### 3.2 Patients with TIO and XLH have distinct metabolic profiles

**Fig 1:**
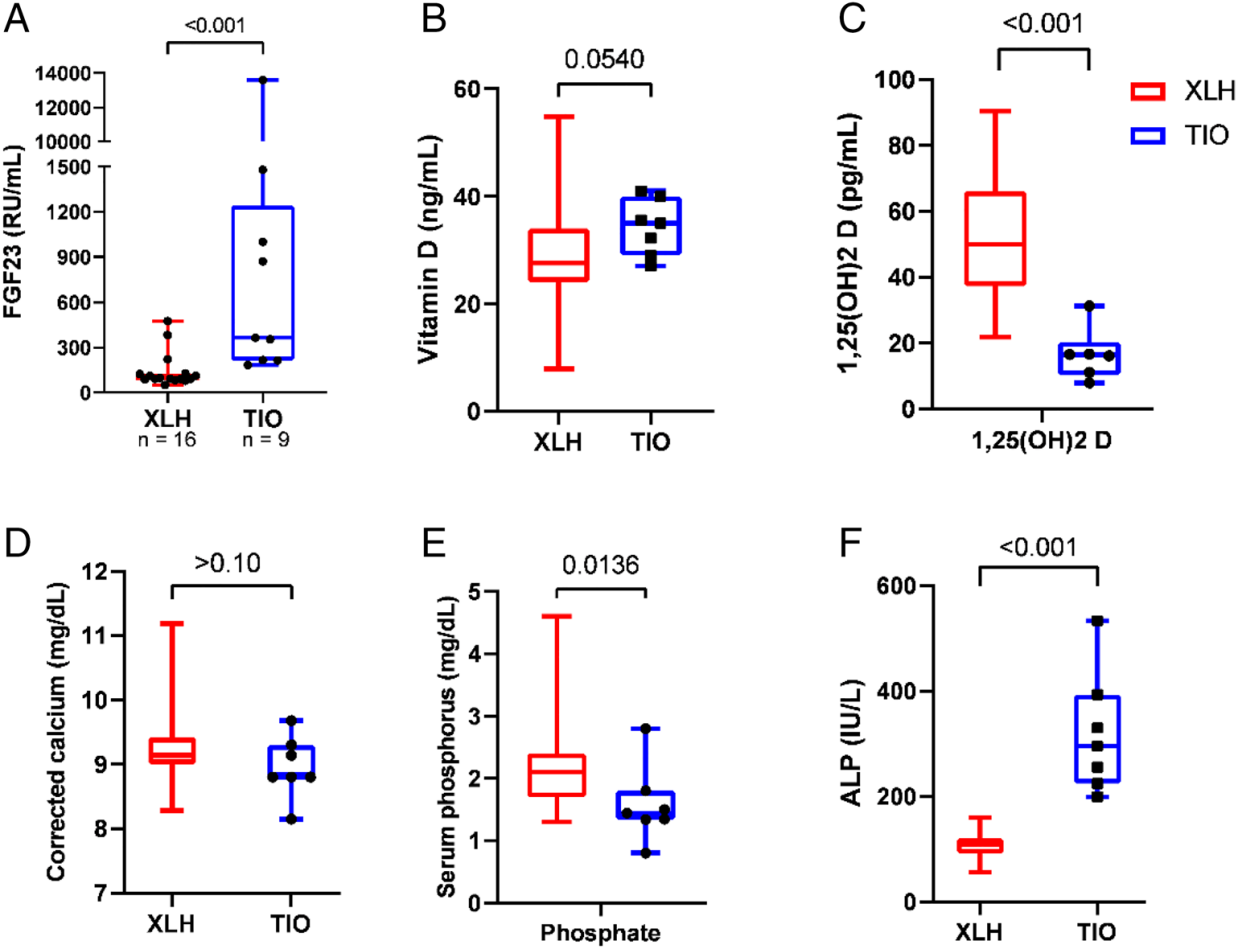
(*A–F*) Comparison of biochemical data between adult patients with tumor‐induced osteomalacia (TIO) and X‐linked hypophosphatemia (XLH). Median, interquartile range, range, and *p* value are displayed. FGF23 = fibroblast growth factor 23; ALP = alkaline phosphatase.

Complete metabolic panel data, along with serum FGF23, phosphorus, PTH, and vitamin D panels, were compared between cohorts by Wilcoxon rank‐sum test. FGF23 values were resulted for all TIO patients but only 37.2% of our XLH patients. FGF23 concentrations were significantly higher in TIO than in XLH (365 versus 95 RU/mL, *p* < 0.001; NR < 180 RU/mL; Fig. 1*A*).

While serum total vitamin D was similar between cohorts, 1,25(OH)_2_ D was significantly reduced in the TIO cohort (16.6 versus 59.8 pg/mL, *p* < 0.001; NR 19.5 to 67.0 pg/mL), potentially a consequence of inhibition of 1a‐hydroxylase by FGF23 (Fig. 1*B*, *C*). The TIO cohort also had similar corrected calcium (8.80 versus 9.14 mg/dL, *p* > 0.10; NR 8.6 to 10.3 mg/dL), reduced serum phosphorus (1.4 versus 2.2 mg/dL, *p* = 0.014; NR 3.4 to 4.5 mg/dL), and elevated alkaline phosphatase (282.8 versus 118.5 IU/L, *p* < 0.001; NR 44 to 147 IU/L) compared with the XLH cohort (Fig. 1*D–F*). Both groups had similarly elevated PTH concentrations (84 versus 83 pg/mL, *p* > 0.10; NR 11 to 51 pg/mL), with 91% (39/43) of XLH patients and 89% (8/9) of TIO patients having PTH values above the normal range. Interestingly, only 22% and 14% of these patients, respectively, had a past diagnosis of hyperparathyroidism. These findings highlight the varying etiologies of XLH and TIO and support the use of CMP, phosphorus, and vitamin D panels in their differentiation.

### 3.3 Bone density, osteoporosis, fractures, and height

**Fig 2:**
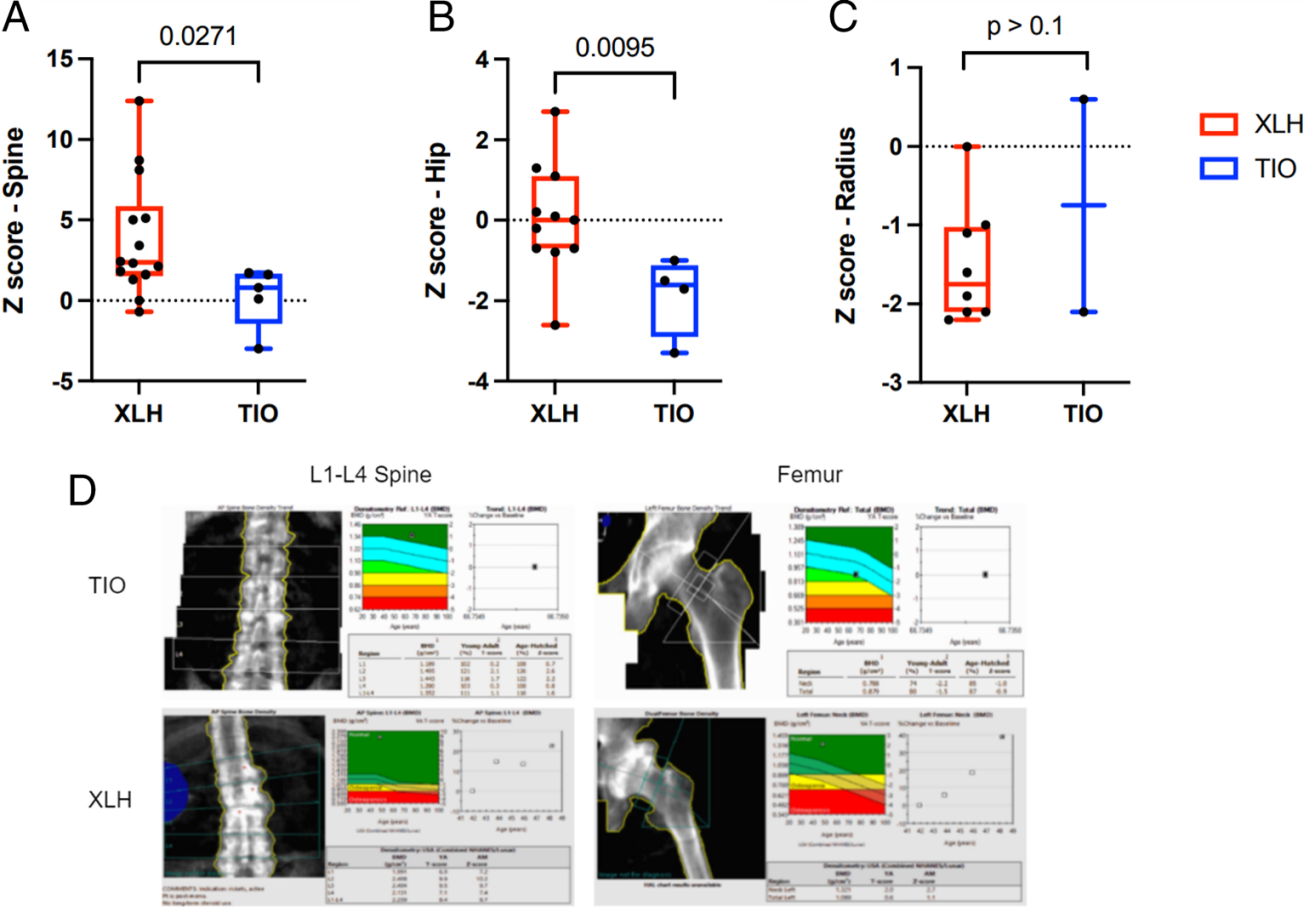
(*A–C*) Bone mineral density analysis of hip, spine (L_1_ to L_4_), and radius (lower 1/3) in patients with X‐linked hypophosphatemia (XLH) and tumor‐induced osteomalacia (TIO). Median, interquartile range, range, and *p* value are displayed. (*D*) Representative dual‐energy X‐ray absorptiometry (DXA) bone density scans from L_1_ to L_4_ spine (left column) and left femur (right column) of patients with TIO and XLH.

Given these profound differences in biochemical profiles, we hypothesized that the two groups would have differences in bone mineral density (BMD) as measured by DXA scan. DXA scans of the hip, spine, and radius were completed in 44% and 26%, 56% and 33%, and 33% and 16% of our TIO and XLH patients, respectively. All patients were adults older than 25 years at time of measurement and so had likely completed somatic skeletal growth. Analysis of DXA scans revealed that TIO patients had lower *Z*‐scores in the hip (−1.6 versus 0.050, *p* = 0.0095) and lumbar spine (0.80 versus 2.35, *p* = 0.0271) but similar *Z*‐scores in the radius (−0.750 versus −1.350, *p* > 0.10) when compared with the XLH cohort (Fig. 2*A–C*). These differences in BMD translated to a high incidence of BMD‐diagnosed osteoporosis in the TIO cohort (6/9, 66.7%) but an absence of the disease in the XLH cohort. In contrast, paraspinal calcifications are common in XLH and likely contribute to our cohort's paradoxically elevated lumbar spine BMDs, as well as the high incidence of spinal stenosis (5/43, 11.6%), which was absent in the TIO cohort. Representative images of bone scans from each cohort are presented in Fig. 2*D*.

### 3.4 TIO and XLH differentially impact bone and dental health

We next examined potential consequences of the differences in bone health between TIO and XLH patients, such as pain, fracture history, and mobility. Both TIO and XLH groups had high incidence of arthralgias (5/9, 56%; 16/43, 37%, *p* > 0.10) and osteoarthritis (44% versus 33%, *p* > 0.10). TIO patients had sustained an average of 8 fractures each, most frequently in the rib, hip, and femur, whereas fractures were rare in XLH (5 fractures total in cohort). Further, 3 patients had pseudofractures in the XLH cohort, whereas no pseudofractures were present in the TIO group.

In contrast to the severe TIO fracture burden, XLH patients had far more abnormalities in secondary dentition, including prior extractions, abscesses, root canals, and implant rejections. A total of 95% (33/35) of XLH patients with recorded dental histories had abnormalities in secondary dentition, with a median of 4 problematic teeth per patient. Just 44% (4/9) TIO patients had secondary dental abnormalities (*p* < 0.001).

Interestingly, we found that TIO patients were taller at the beginning of their disease than XLH patients but lost this height difference by time of resection (median height loss 1.5 cm). Further, 100% of the TIO patients reported using assistive devices such as a cane, walker, or wheelchair for daily living compared with just 14% (6/43) of the XLH patients.

Finally, these patients also differed in incidence and types of corrective surgeries. Among XLH patients, 19% (8/43) had undergone osteotomies, whereas no TIO patients had. These osteotomies are likely to correct the bowing deformities characteristic of XLH. On the other hand, TIO patients were more likely to have had knee replacements (33% versus 7%, *p* = 0.024) but not hip replacements (22% versus 14%, *p* = 0.53).

## 4 Discussion

TIO is a rare paraneoplastic phosphate‐wasting syndrome with a high rate of misdiagnosis, owing to small tumor size and similar clinical presentation to more common phosphate‐wasting disorders such as XLH. Importantly, TIO patients acquire pathologies quickly after initial symptom onset, so timely diagnosis is essential to minimizing health burden on the patient.

In this retrospective analysis, we validated cohorts of patients with TIO and XLH using rigorous inclusion criteria and identified the salient clinical features that best distinguish them. TIO diagnoses were confirmed via location, removal, and histologic identification of a phosphaturic mesenchymal tumor. XLH diagnoses were confirmed via the presence of a PHEX gene mutation. The genetic tools and scans used to confirm TIO and XLH are new relative to the literature on these diseases. As such, much literature in the past relied on clinical diagnoses, which introduces the possibility of error.^(^ ^4^ ^)^ Using these methods, we identified common biochemical and clinical variables that meaningfully distinguish TIO from XLH. A summary of the distinguishing biochemical and clinical features is provided (Table 2).

**Table 2:** Summary of Clinical Differentiators Between X‐Linked Hypophosphatemia (XLH) and Tumor‐Induced Osteomalacia (TIO) Resolved in This Study

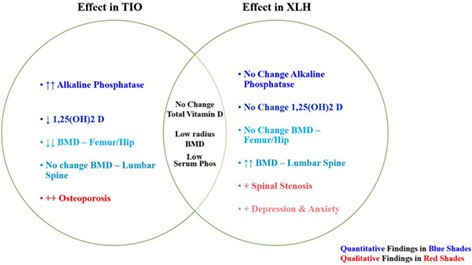

Arrows denote direction above (↑, ↑↑) or below (↓, ↓↓) the value's normal limits, with more arrows indicating a more profound effect. Dash indicates no difference from normal limits. Likewise, plus signs (+, ++) indicate disease presence in the cohort. NA = not applicable, disease not found in indicated cohort. “Osteoporosis” denotes the clinical diagnosis.

We first examined differences in laboratory abnormalities associated with our TIO and XLH cohorts. Both groups had normal total vitamin D, but TIO patients had significant reductions in serum 1,25(OH)_2_ D compared with patients with XLH. The TIO cohort also had significantly reduced serum calcium and phosphorus, as well as significantly elevated alkaline phosphatase. Both cohorts are higher than normal median BMIs. There are some hypotheses that the metabolic derangement or physical inactivity observed in XLH (and TIO) could lead to elevations in BMI, but further studies are needed to probe this claim.^(^ ^16^, ^17^, ^18^ ^)^

**Fig 3:**
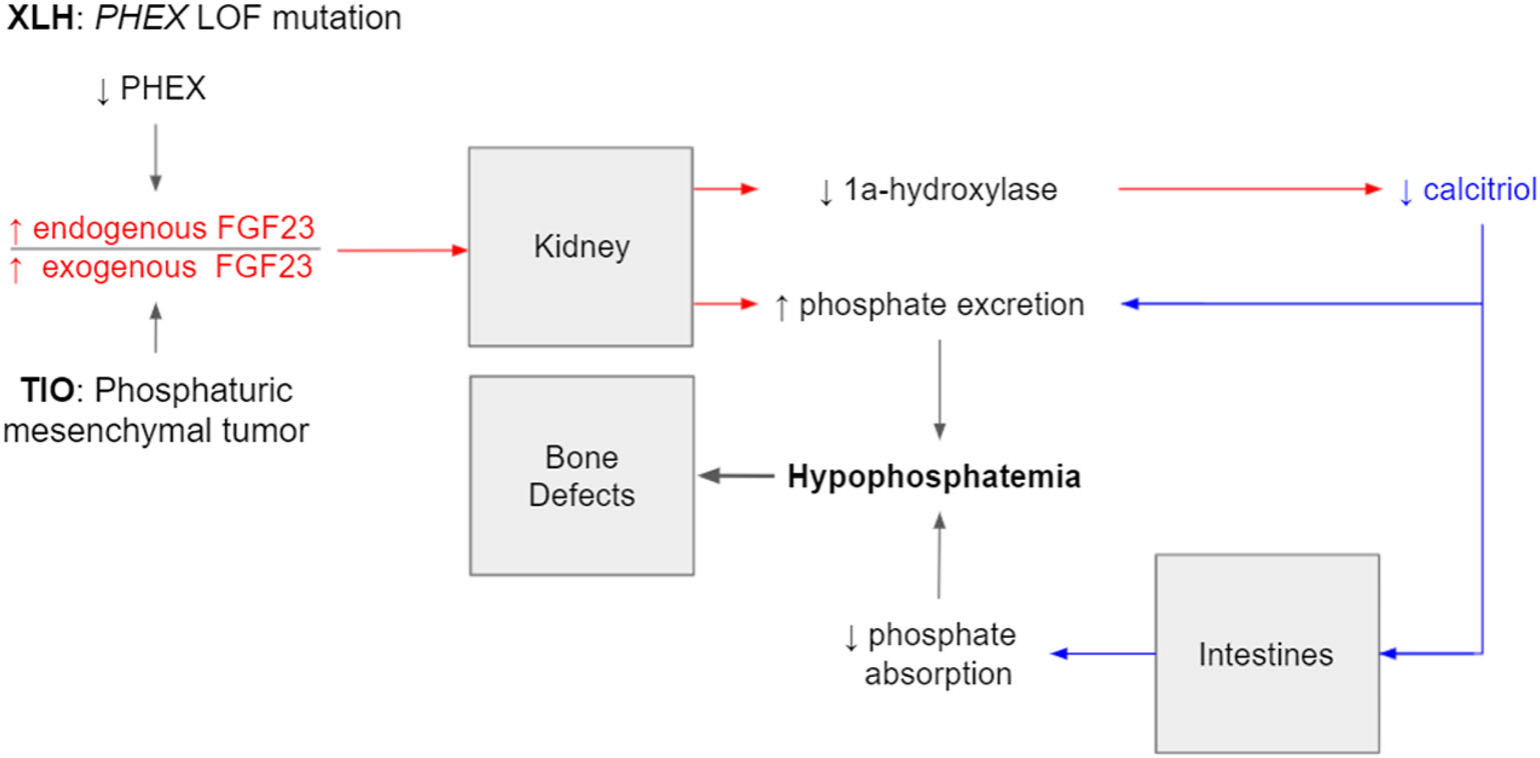
Effect of FGF23 and 1,25(OH)_2_ D (calcitriol) on bone metabolism in tumor‐induced osteomalacia (TIO) and X‐linked hypophosphatemia (XLH). Calcitriol is a critical metabolite in regulating calcium and phosphate absorption in the intestine and kidney. Bench studies have demonstrated that FGF23 acts on the kidney both to decrease phosphate reabsorption in the proximal tubule and to inhibit 1a‐hydroxylase, the enzyme responsible for the activation of vitamin D to calcitriol. By contrast, XLH is an X‐linked dominant phosphate‐wasting disorder caused by a loss‐of‐function mutation in the phosphate‐regulating neutral endopeptidase homolog X‐linked (*PHEX*) gene, which causes elevated levels of endogenously produced serum FGF23.

A detailed mechanism of the effects of TIO and XLH on bone metabolism is provided in Fig. 3. Briefly, PMTs in TIO ectopically secrete FGF23, whereas native osteocytes oversecrete FGF23 in XLH due to a loss of function in the PHEX protein. Elevated FGF23 dysregulates bone homeostasis through its effect on phosphate and calcium absorption by the intestine and wasting by the kidney. While mechanistically similar, we demonstrate in this study that PMTs in TIO elevate FGF23 significantly more than endogenous overproduction does in XLH.^(^ ^12^ ^)^ Notably, only 37.2% (16/43) of our XLH patients had FGF23 measured, as practice patterns have evolved in recent years to more efficiently use genetic panels to test for a positive *PHEX* mutation in patients with high suspicion for XLH (eg, positive family history).

In TIO, these metabolic disturbances increase bone turnover, precipitating in the observed high ALP and numerous fractures. In contrast to the adult onset typical of TIO, phosphate wasting begins early in life in patients with XLH, causing characteristic bowed legs, rickets/osteomalacia, and frequent dental issues in adulthood. Our analysis also revealed that PTH is elevated in both TIO and XLH but that a small fraction of these people have been diagnosed with secondary hyperparathyroidism.

These biochemical disturbances correlated with valuable clinical insights. We found that TIO patients may become shorter over the course of their disease, stressing the importance of early, accurate diagnosis of TIO. Overall bone health also differed greatly between cohorts, with TIO patients having significantly lower femur/hip BMDs than both XLH patients and healthy population. By consequence, osteoporosis, fracture, and knee arthroplasty incidences were much higher in the TIO cohort, which agrees with prior studies of TIO.^(^ ^10^ ^)^ Importantly, osteomalacia and rickets are both known causes of pseudofractures, which are areas of low‐density bone and thickened periosteum on imaging. While pseudofractures have been identified in both TIO and XLH, our cohorts only demonstrate their presence in XLH; however, this may be an artifact of low sample size.^(^ ^8^, ^19^ ^)^

Our XLH patients also had a markedly higher burden of abnormalities in secondary dentition (95%) compared with both our TIO patients (44%) and frequencies of dental pathologies for *PHEX*‐mutation confirmed XLH (63%) reported by Chester and colleagues.^(^ ^20^ ^)^ Our data likely depart from literature because we liberally define these abnormalities as “one or more secondary tooth extraction, dental abscess, or root canal,” whereas Chester and colleagues restricted their scope to dental disease.

Interestingly, our TIO patients had similar lumbar spine BMDs to healthy populations, which differs from those described in previous TIO literature, a fact that may be attributable to the lower median time‐to‐diagnosis and small sample size of our cohort.^(^ ^21^ ^)^ On the other hand, XLH patients had significantly higher BMDs than healthy controls and TIO patients. XLH has been demonstrated to cause calcifications of paraspinal ligaments, leading to paradoxically high lumbar spine BMDs in a hypophosphatemic disease.^(^ ^22^ ^)^ Indeed, our XLH patients had a high incidence of spinal stenosis, whereas the TIO cohort had no such incidence, likely attributable to the observed lumbar calcification. Although it is likely that these paraspinal calcifications inflate the lumbar‐spine *Z*‐scores presented here, this phenomenon should be studied more thoroughly with tandem plain films of the spine. These findings may be particularly useful for orthopedic surgeons when evaluating surgery candidates for underlying conditions.

The age at symptom onset is an important distinction to emphasize between these two cohorts. XLH patients are born into disease and are vulnerable to rickets due to affected bone modeling throughout skeletal development, whereas TIO often occurs after growth‐plate closure and causes osteomalacia through effects on bone remodeling. With one exception, all patients in the TIO cohort presented here were >25 years old at symptom onset and are assumed to have reached skeletal maturity. The one exception had symptoms beginning at age 18 years but was misdiagnosed with XLH for 26 years, thus accruing a spectrum of symptoms from both rickets (eg, short stature) and osteomalacia (eg, fractures) over the disease course. Interestingly, although this patient did have more total complications than other TIO patients due to length of disease, the patient's metabolic profile and bone densities did not differ significantly from the other TIO patients in our cohort.

This investigation of XLH and TIO in adults complements a recent retrospective comparison of the presentations of XLH and tumor‐induced osteomalacia/rickets in a cohort of children in China.^(^ ^10^ ^)^ In agreement with our results, this study also identified serum phosphorus, serum ALP, FGF23, and lumbar spine BMD as important differentiators of the two diseases in pediatric patients. Interestingly, the authors found no incidence of dental problems in their pediatric TIO cohort, whereas 22% of our TIO patients were edentulous. Hence, tooth decay/loss may be a relevant symptom in adults but not children with TIO, but no study to our knowledge has examined how TIO symptoms vary by age of onset. The authors also found high incidence of rickets‐specific symptoms in pediatric TIO patients, whereas the symptoms present in our adult TIO cohort (eg, fractures) are typical of osteomalacia. This difference is likely reflective of bone immaturity in pediatric patients. Finally, the authors found significantly higher incidence of lower‐extremity weakness in TIO patients. Although we did not examine weakness or fatigue directly in this study, recent studies have demonstrated that severe fatigue is common in adults with TIO.^(^ ^10^, ^21^ ^)^

Our study benefits from the certainty of diagnosis of each patient, but a drawback to this approach is low statistical power, which may have prevented us from identifying other, less evident variables. Additionally, though literature has mixed findings regarding the prevalence of XLH by sex, some groups suggest a slight a male bias in XLH prevalence due to X‐inactivation, whereas our cohort was only 28% male.^(^ ^20^, ^23^, ^24^ ^)^ Although this introduces the possibility of error due to sex differences, studies with *PHEX*‐mutation confirmed XLH have not identified such an effect.^(^ ^24^, ^25^ ^)^ This study also carries the same limitations of any retrospective analysis, such as ascertainment and recall bias, which may be implicated in the identification of XLH patients in the database used for this study.^(^ ^26^ ^)^ As such, we recommend using the results of this study only to supplement standard diagnostic procedures until they are validated in a prospective, multicenter study. Additionally, missing CBC and TmP/GFR data from our XLH patients forced us to exclude these data from our analysis. These data may reveal essential differences in the hematological and renal consequences of these diseases, and they should be studied in future works comparing TIO and XLH.

In conclusion, we have presented the first study comparing TIO and XLH in adults and the first study of these diseases in which each diagnosis was adjudicated with genotyping (XLH) or pathologic confirmation of a PMT (TIO). Our results support the use of serum phosphorus, 1,25(OH)_2_ D, a CMP, and DXA bone scans in differentiating TIO and XLH. They also propose height, history of fractures, osteoporosis, spinal stenosis, osteotomies, and depression and anxiety as additional demographic features that should be considered in a patient presenting with phosphate wasting. We recommend these findings be used to augment established clinical reasoning. In the future, prospective studies should be performed to validate the findings of this retrospective analysis, observe measures not studied here (eg, functional studies like timed‐up‐and‐go (TUG) testing), and generate reliable diagnostic algorithms for endocrinologists and orthopedists.

## Conflict of Interest

KD is a clinical trial investigator and consultant for Ultragenyx and Alexion Pharmaceuticals. JAD and ER are supported by NIGMS of the National Institutes of Health under award number T32GM007347. The content in this report is solely the responsibility of the authors and does not necessarily represent the official views of the National Institutes of Health. The remaining authors report no disclosures.

### Peer Review

The peer review history for this article is available at https://publons.com/publon/10.1002/jbm4.10580.

## Data Availability

The data that support the findings of this study are available on request from the corresponding author. The data are not publicly available due to privacy or ethical restrictions.

## References

[1] Martin A , Quarles LD . Evidence for FGF23 involvement in a bone‐kidney axis regulating bone mineralization and systemic phosphate and vitamin D homeostasis. Adv Exp Med Biol. 2012;728:65‐83. 22396162 10.1007/978-1-4614-0887-1_4 PMC6350529

[2] Hoffman WH , Jueppner HW , Deyoung BR , O'Dorisio M , Given KS . Elevated fibroblast growth factor‐23 in hypophosphatemic linear nevus sebaceous syndrome. Am J Med Genet A. 2005;134:233‐236. 15742370 10.1002/ajmg.a.30599

[3] Imel EA , Econs MJ . Fibrous dysplasia, phosphate wasting and fibroblast growth factor 23. Pediatr Endocrinol Rev. 2007;4:(Suppl 4):434‐439. 17982392

[4] Yin Z , Du J , Yu F , Xia W . Tumor‐induced osteomalacia. Osteoporos Sarcop. 2018;4:119‐127. 10.1016/j.afos.2018.12.001 PMC6372818 30775554

[5] Folpe AL . Phosphaturic mesenchymal tumors: a review and update. Semin Diagn Pathol. 2019;36:260‐268. 31301876 10.1053/j.semdp.2019.07.002

[6] Beck‐Nielsen SS , Mughal Z , Haffner D , et al. FGF23 and its role in X‐linked hypophosphatemia‐related morbidity. Orphanet J Rare Dis. 2019;14:58. 30808384 10.1186/s13023-019-1014-8 PMC6390548

[7] Liu S , Guo R , Simpson LG , et al. Regulation of fibroblastic growth factor 23 expression but not degradation by PHEX *. J Biol Chem. 2003;278:37419‐37426. 12874285 10.1074/jbc.M304544200

[8] Skrinar A , Dvorak‐Ewell M , Evins A , et al. The lifelong impact of X‐linked hypophosphatemia: results from a burden of disease survey. J Endocr Soc. 2019;3:1321‐1334. 31259293 10.1210/js.2018-00365 PMC6595532

[9] Feng J , Jiang Y , Wang O , et al. The diagnostic dilemma of tumor induced osteomalacia: a retrospective analysis of 144 cases. Endocr J. 2017;64:675‐683. 28450684 10.1507/endocrj.EJ16-0587

[10] Jiajue R , Ni X , Jin C , et al. Early discrimination between tumor‐induced rickets/osteomalacia and X‐linked hypophosphatemia in Chinese children and adolescents: a retrospective case–control study. J Bone Miner Res. 2021;36(9):1739‐1748. 33971042 10.1002/jbmr.4331

[11] Colazo JM , DeCorte JA , Gillaspie EA , Folpe AL , Dahir KM . Hiding in plain sight: gene panel and genetic markers reveal 26‐year undiagnosed tumor‐induced osteomalacia of the rib concurrently misdiagnosed as X‐linked hypophosphatemia. Bone Rep. 2021;14:100744. 33490314 10.1016/j.bonr.2020.100744 PMC7804981

[12] Dahir K , Roberts MS , Krolczyk S , Simmons JH . X‐linked hypophosphatemia: a new era in management. J Endocr Soc. 2020;4(12):bvaa151. 33204932 10.1210/jendso/bvaa151 PMC7649833

[13] Minisola S , Molinolo AA , Chen CC , Collins MT . Tumour‐induced osteomalacia. Nat Rev Dis Primers. 2017;3:17044. 28703220 10.1038/nrdp.2017.44

[14] Carpenter TO . New perspectives on the biology and treatment of X‐linked hypophosphatemic rickets. Pediatr Clin North Am. 1997;44:443‐466. 9130929 10.1016/s0031-3955(05)70485-5

[15] Stekhoven DJ , Bühlmann P . MissForest—non‐parametric missing value imputation for mixed‐type data. Bioinformatics. 2012;28:112‐118. 22039212 10.1093/bioinformatics/btr597

[16] Haffner D , Emma F , Eastwood DM , et al. Clinical practice recommendations for the diagnosis and management of X‐linked hypophosphataemia. Nat Rev Nephrol. 2019;15:435‐455. 31068690 10.1038/s41581-019-0152-5 PMC7136170

[17] Simpson CA , Santoro AM , Carpenter TO , Insogna KL . Serum levels of Lipocalin are lower in adolescents with X‐linked hypophosphatemia. J Endocr Soc. 2021;5:A27. 10.1210/jendso/bvad116 PMC10583534 37860221

[18] Zhukouskaya VV , Jauze L , Charles S , et al. Increased prevalence of overweight and obesity in children with X‐linked hypophosphatemia. Endocr Connect. 2020;9:144‐153. 31910157 10.1530/EC-19-0481 PMC6993252

[19] Jan de Beur SM , Miller PD , Weber TJ , et al. Burosumab for the treatment of tumor‐induced osteomalacia. J Bone Miner Res. 2021;36:627‐635. 33338281 10.1002/jbmr.4233 PMC8247961

[20] Chesher D , Oddy M , Darbar U , et al. Outcome of adult patients with X‐linked hypophosphatemia caused by PHEX gene mutations. J Inherit Metab Dis. 2018;41:865‐876. 29460029 10.1007/s10545-018-0147-6 PMC6133187

[21] Jerkovich F , Nuñez S , Mocarbel Y , et al. Burden of disease in patients with tumor‐induced osteomalacia. JBMR Plus. 2020;5(2):e10436. 33615105 10.1002/jbm4.10436 PMC7872334

[22] Beck‐Nielsen SS , Brusgaard K , Rasmussen LM , et al. Phenotype presentation of hypophosphatemic rickets in adults. Calcif Tissue Int. 2010;87:108‐119. 20524110 10.1007/s00223-010-9373-0

[23] Hardy DC , Murphy WA , Siegel BA , Reid IR , Whyte MP . X‐linked hypophosphatemia in adults: prevalence of skeletal radiographic and scintigraphic features. Radiology. 1989;171:403‐414. 2539609 10.1148/radiology.171.2.2539609

[24] Ruppe M . X‐linked hypophosphatemia. In Adam HA , Pagon RA , eds. GeneReviews [Internet]. Seattle: University of Washington; 2017.

[25] Yamamoto A , Nakamura T , Ohata Y , Kubota T , Ozono K . Phenotypes of a family with XLH with a novel PHEX mutation. Hum Genome Variat. 2020;7:8. 10.1038/s41439-020-0095-1 PMC7109063 32257293

[26] Mantel N , Haenszel W . Statistical aspects of the analysis of data from retrospective studies of disease. J Nat Cancer Inst. 1959;22:719‐748. 13655060

